# Clinical review and drug sensitivity test of *Corynebacterium kroppenstedtii* complex isolates in non-lactating patients with severe mastitis

**DOI:** 10.3389/fmicb.2024.1501204

**Published:** 2024-12-23

**Authors:** Zuxin Liang, Zhujun Zeng, Yanqiang Liao, Yaxin Cao, Dongmei Li, Ningbo Deng, Yeyan Lei, Xuanhui Long, Chenguang Shen, Rui Xu

**Affiliations:** ^1^BSL-3 Laboratory (Guangdong), Guangdong Provincial Key Laboratory of Tropical Disease Research, School of Public Health, Department of Laboratory Medicine, Zhujiang Hospital, Southern Medical University, Guangzhou, China; ^2^Department of Laboratory Medicine, Guangdong Provincial Hospital of Chinese Medicine, Zhuhai, China; ^3^Department of Laboratory Medicine, Doumen Qiaoli Hospital of Traditional Chinese Medicine, Zhuhai, Guangdong, China; ^4^Tianjin Medical Device Evaluation and Inspection Center, Tianjin, China; ^5^Department of Breast, Guangdong Provincial Hospital of Chinese Medicine, Zhuhai, China; ^6^Key Laboratory of Infectious Diseases Research in South China (Southern Medical University), Ministry of Education, Guangzhou, China

**Keywords:** non-puerperal mastitis, mastitis, *Corynebacterium kroppenstedtii* complex, antibiotic susceptibility test, daptomycin

## Abstract

**Background:**

Previous microbiological investigations have demonstrated a significant correlation between *Corynebacterium kroppenstedtii* complex (CKC) infection and mastitis. Recent studies have confirmed the existence of the CKC, with *Corynebacterium parakroppenstedtii* (*C. parakroppenstedtii*) identified as the primary infectious agent. Examining the incidence of CKC in cases of severe non-lactational mastitis, alongside the clinical characteristics of infected patients, as well as evaluating the drug sensitivity testing protocols for CKC, can provide a more robust foundation for the diagnosis and treatment of CKC infections.

**Methods:**

Data regarding the diagnosis and treatment of non-nursing patients with severe mastitis who underwent surgical intervention were extracted from the hospital’s electronic medical record system. Additionally, drug susceptibility tests were conducted on 15 strains of CKC isolated from mammary abscesses as well as DSM 44385 model strains. The effects of β-NAD and Tween80 (TW80) on the antibiotic susceptibility test by AGAR dilution and micro broth dilution were analyzed.

**Results:**

In this study, *C. parakroppenstedtii* accounted for 80% (12/15) of the isolates, while *Corynebacterium pseudokroppenstedtii* made up 13.3% (2/15), and *Corynebacterium kroppenstedtii* was identified in only 6.7% (1/15) of the cases. There were significant differences in age at first delivery (*p* < 0.001), prolactin (*p* < 0.001), CRP (*p* < 0.05), WBC (*p* < 0.05), and NEUT (*p* < 0.05) between CKC positive group and CKC negative group. In the AGAR dilution test, the addition of β-NAD only caused acceptable differences in penicillin G and ciprofloxacin but did not affect 12 antibiotics. There are 14 drugs with good coincidence rates (92.9%) in the micro broth dilution method and agar dilution method without the addition of β-NAD. The addition of 0.05% (v/v) TW80 resulted in all strains being resistant to penicillin G. Daptomycin is not suitable for the micro broth dilution method.

**Conclusion:**

Elderly primiparas with high prolactin levels have a higher risk of CKC infection. The micro broth dilution method is not applicable for EUCAST drug susceptibility testing for CKC and there is no suitable drug susceptibility evaluation procedure for daptomycin against CKC.

## 1 Introduction

Non-puerperal mastitis (NPM) is a group of benign, non-specific inflammatory diseases of unknown etiology, and its diagnosis lacks a gold standard. It is usually a comprehensive diagnosis based on pathology combined with clinical manifestations and auxiliary examination, excluding breast tuberculosis, specific granulomatous lesions, and cancer. Bacterial infection is one of the risk factors for NPM, and studies have confirmed that the mammary gland has a different microecology in the absence of inflammation, and benign or malignant lesions (Hieken et al., 2016). Still, the role of microorganisms in the occurrence and development of NPM remains to be clarified. Previous research suggested that infections caused by *Corynebacterium kroppenstedtii (C. kroppenstedtii)* might be associated with the prevalence of mastitis. However, Luo et al. (2022) reported that *Corynebacterium parakroppenstedtii sp. nov (C. parakroppenstedtii)* is the primary strain detected in mastitis cases, followed by *Corynebacterium pseudokroppenstedtii sp. nov (C. pseudokroppenstedtii)*. Liu et al. found that *C. kroppenstedtii-*like strains could be divided into five species, rather than the previously reported three species, with minimal evolutionary differences. And *C. parakroppenstedtii* secretes a novel glycolipid that promotes inflammation in granulomatous lobular mastitis (GLM) (Liu et al., 2024). Existing studies confirm that the proportion identified as *C. kroppenstedtii* is actually minimal (Liu et al., 2024). Referring to this group as the *Corynebacterium kroppenstedtii* complex (CKC) better summarizes these findings (Huang et al., 2024). Therefore, in subsequent references to earlier literature, using CKC instead of *C. kroppenstedtii* aligns more accurately with the current finding. In China, a significant number of non-lactating mastitis patients have been found to be infected with CKC, indicated that the correlation between CKC infection and mastitis in this population is not uncommon when compared to the atypical definition of breast infections observed in white women (Paviour et al., 2002; Dobinson Hazel et al., 2015). However, the underlying pathological mechanisms linking CKC to mastitis are still being investigated. Nevertheless, antibiotic therapy informed by drug susceptibility testing has become a crucial component of disease management strategies (Dobinson Hazel et al., 2015).

We retrospectively collected and analyzed clinical information of non-lactating mastitis patients who underwent breast segment resection and surgical pus discharge in our hospital and tested the drug resistance phenotype of 15 strains of CKC stored in accordance with CLSI and EUCAST procedures. To explore the applicability of these executive procedures in CKC drug susceptibility test.

## 2 Materials and methods

### 2.1 Patients and clinical samples

A total of 77 inpatients diagnosed with non-lactating mastitis who underwent surgical resection of the breast between May 2019 and December 2022 were reviewed using the hospital’s electronic medical record system. Patient demographics and laboratory test results were analyzed, while cases of breast cancer, lipoma, and breast tuberculosis were excluded from the study. According to the results of CKC culture, enrolled patients were divided into the following three groups: Group A consisted of patients with positive CKC cultures, while Group B included those with negative cultures (comprising sterile growth and non-CKC growth). Patients without any culture records were classified as Group C.

### 2.2 Bacterial cultivation and identification

The bacteria were inoculated with standard inoculation media (BAP, plain chocolate, and MacConkey plates) and BAP plates impregnated with 1 ml 1% TW80 (v/v), culture at 35°C, 5% CO_2_ for 3–5 days. The colonies were identified by mass spectrometry using French Merieux VITEK MS.

### 2.3 DNA sequencing and analysis

Genomic DNA was extracted using a bacterial genomic DNA extraction kit (AG, China) according to the manufacturer’s instructions. All clinical isolates underwent partial sequencing of the *16S rRNA*, *rpoB*, and *fusA* genes (Weisburg et al., 1991; Khamis et al., 2004; Busse et al., 2019). The primers are shown in the Supplementary Table 1. Sequences were compared with type strains DSM 44385 on the MegAlign. The phylogenetic tree was constructed using the neighbor-joining method in MEGA (version X) software with 1,000 bootstrap repetitions.

### 2.4 Drug susceptibility testing

A total of 15 strains of CKC and type strain DSM 44385 isolated from non-lactating mastitis patients were tested by the AGAR dilution method and micro broth method. Duplicate detections were eliminated. EUCAST and CLSI do not provide specific terms for the susceptibility testing of CKC, so this study refers to the susceptibility testing requirements of the genus *Corynebacterium*. The micro broth dilution method is endorsed in EUCAST Clinical Breakpoint Tables v.13.0 and CLSI-M45, while the preparation of AGAR plates in the AGAR dilution method is recommended by CLSI-M07 11th Edition. CLSI-M45 recommends 17 kinds of drugs, while EUCAST recommends 9 kinds of drugs. With reference to the former, we selected 15 kinds of 13 categories, which covered the types of drugs of the latter. The source of the drug is the standard grade of China National Institute of Food and Drug Control.

The drug dilution concentration design of AGAR dilution method and Minimum Inhibitory Concentration (MIC) method is as follows: 12 concentrations of cefepime, penicillin G, ceftriaxone, ciprofloxacin, rifampicin, linezolid, Clindamycin, vancomycin, meropenem and daptomycin were set (32 μg/mL dilution to 0.015 μg/mL); 12 concentrations of tetracycline and doxycycline were set (64 μg/mL dilution to 0.03 μg/mL); Erythromycin was set at 9 concentrations (8 μg/mL dilution to 0.03 μg/mL); Four concentrations of cotrimoxazole were set (1/19 μg/mL, 2/38 μg/mL, 4/76 μg/mL, 8/152 μg/mL).

The EUCAST procedure required the addition of “20 mg/L β-NAD” as a supplement in the micro broth dilution method, but the CLSI procedure did not. The AGAR dilution method does not require the addition of β-NAD. In this study, two groups with and without the addition of β-NAD were also set up to observe the effect of the addition of β-NAD on the AGAR dilution method. The Daptomycin drug susceptibility test is only recommended for microdilution of broth.

According to the CLSI-M100 file, *Escherichia coli* ATCC 25922 was used for quality control of the gentamicin group without β-NAD, and *Streptococcus pneumoniae* ATCC 49619 was used for the quality control of other drugs with the β-NAD group. There is no corresponding quality control standard for other combinations.

The CLSI micro broth dilution *Corynebacterium* breakpoint was used as the standard. CLSI provides micro broth dilution for all 15 drugs. EUCAST offers 8 drugs breakpoints for micro broth dilution, excluding tetracycline, ciprofloxacin, penicillin G, and gentamicin. The AGAR dilution method does not have a file providing a breakpoint standard, so we chose to refer to the breakpoint standard data of the CLSI micro broth dilution method.

To evaluate the impact of TW80 on the drug sensitivity assessment using the micro broth dilution method for penicillin G, this study opted not to include β-NAD and instead incorporated 0.05% (v/v) TW80 in the testing of penicillin G.

The drug sensitivity testing incubation environment for this study was 35°C, 5% CO_2_, and drug sensitivity interpretation was performed after 36 h of testing. The drug sensitivity test has three possible outcomes: when the results are the same, it is judged as “Complete consistency”; when the results are “resistance (R)” and “sensitivity (S)”, it is judged as “inconsistency”, indicating the presence of “false sensitivity” or “false resistance”, which is an unacceptable outcome. When the comparison results are “I and S” or “I and R” respectively, it is judged as “partial consistency” because the drug sensitivity test is a qualitative test of dilution ratio, and a deviation of one concentration gradient is considered an acceptable error. S, I, and R are usually distributed in three consecutive values, and the I value can be allowed to fluctuate between S/R.

### 2.5 Follow-up investigation

The patients who were discharged >12 months prior were visited by telephone to investigate the wound healing, whether the symptoms of redness, swelling, and pain disappeared, whether there was a lump on palpation, and the results of color ultrasound.

### 2.6 Statistical analysis

Laboratory results between the groups were compared using the Mann–Whitney U test (Que et al., 2024). A *p*-value of <0.05 was considered statistically significant.

### 2.7 Ethical statement

This investigation is a non-prospective study, and the Ethics Committee of Guangdong Provincial Hospital of Traditional Chinese Medicine exempted this project from ethical review.

## 3 Results

### 3.1 Epidemiological characteristics, clinical manifestations, pathological changes, and serum testing of the population

Microbial culture records were found in 55 of 77 patients with breast segmentectomy, and CKC was detected in 47.3% (26/55, group A). There were 29 culture records in group B, including 7 records with other bacteria detected and 22 records with no bacteria detected. Other bacteria included: *Enterobacter aerogenes*, *Staphylococcus epidermidis*, *Citrobacter diversus*, *Streptococcus salivarius*, *Escherichia coli, Corynebacterium agaricum*, *Propionibacterium avarice* and *Propionibacterium azolicum* mixed growth: 1 case each. Group C (*n* = 22) was deficient in culture records.

Group A: 1 case of schizophrenia, 1 case of blunt trauma history, 1 case of syphilis, 1 case of hepatitis B carrier, 3 cases of abnormal thyroid function detection (at least one index of FT3, FT4, TSH, A-TG, TG, A-TPO is abnormal, a total of 10 cases of examination records); In group B, thyroid function was abnormal in 1 case (9 cases were recorded), and hepatitis B was carried in 1 case. The above disease records are missing for Group C.

For concomitant diagnoses in pathological classification, Group A had 8 cases of “local abscess”, 3 cases of “chronic suppurative inflammation”, and 1 case of “fibroadenoma with cystic breast disease”; Group B had 3 cases of “fibroadenoma with cystic breast disease”, 5 cases of “chronic suppurative inflammation”, 3 cases of “local fibroadenoma like hyperplasia”, and 1 case of “fibrocystic breast disease”; Group C had 9 cases accompanied by “local abscess”, 1 case each of “fibrocystic breast disease” and “fibroadenoma like hyperplasia” (Table 1).

**TABLE 1:** Demographic information, clinical manifestation, laboratory tests and pathologic diagnosis.

	Pus culture for fungi and bacteria		
**Characteristic**	**Group A no. (%)**	**Group B no. (%)**	**Group C no. (%)**
All patients	26 (100%)	29 (100%)	22 (100%)
Race (the Han nationality)	26 (100%)	29 (100%)	22 (100%)
Age, mean (range)	33 (19–40)	31.8 (23–44)	37 (25–48)
**Lesion site**			
Right breast	13 (50%)	12 (41.4%)	11 (50%)
Left breast	10 (38.5%)	17 (58.6%)	8 (36.4%)
Bilateral	3 (11.5%)	0	3 (13.6%)
The age of initial birth *	28.2 (24–36)	23.84 (19–28)	24.6 (20–32)
**Parity**			
0	3/26	3/29	2/22
1	16/26	12/29	13/22
2	6/26	8/29	4/22
3	1/26	4/29	3/22
>3	0/26	2/29	0/22
**History of lactation in months**			
0	3/26	6/29	4/22
<6	1/26	4/29	8/22
6–12	18/26	13/29	9/22
>12	4/26	6/29	1/22
**Smoking history**			
Active smoking	0	0	0
Passive smoking	11/26	10/29	6/15
**Blood work**			
WBC mean × 10^9^/L[range]#	10.02 (4.82–15.72)	7.42 (4.13–14.17)	6.33 (4.82–12.61)
NEUT mean × 10^9^/L[range]#	7.52 (2.96–11.33)	5.64 (2.51–12.05)	5.02 (2.61–10.92)
LYMPH, mean × 10^9^/L[range]	1.81 (0.92–3.51)	1.85 (1.16–2.98)	1.62 (1.04–2.35)
CRP, mean mg/L[range]#	19.12 (1–58.1), *n* = 16	5.39 (0.24–13), *n* = 15	4.25 (1–11),*n* = 4
Elevated prolactin level#	674.52 ± 619.0, *n* = 25	470.77 ± 462.35, *n* = 27	172.85 ± 89.75, *n* = 6
**Breast tissue pathology results**			
GM: granulomatous mastitis	22	20	12
GLM: granulomatous lobular mastitis	3	6	6
PCM: plasma cell mastitis	1	1	1
PM: periductal mastitis	0	1	1
CSI: chronic suppurative inflammation	0	1	2

Group A: CKC culture positive; Group B: CKC culture negative/culture negative/other bacterial growth; Group C: Lack of cultivation results;Passive smoking:Family members who live together have active smokers;

“*” indicated that there is a significant difference (*p* < 0.001) between Group A and Group B in the age of initial birth;

“#” indicated that there is a significant difference in this item between group A and group B: WBC (*p* < 0.05), NEUT (*p* < 0.05) CRP (*p* < 0.05), PRO (*p* < 0.001).

### 3.2 Telephone survey

In the telephone follow-up, one patient in group A had intermittent dull pain, one patient in group B had pain, and the other patient had palpation and color ultrasound indicating the possibility of recurrence. In group C, there was one case with slight pain and redness in the wound, no mass was palpated, and no abnormal findings were found by color ultrasound echo. Most patients had no discomfort and gave up the follow-up ultrasound examination (Table 2).

**TABLE 2:** Telephone follow-up results.

	Group A no. (%)	Group B no. (%)	Group C no. (%)
Out of contact	1	2	2
Wound healing	25/25	27/27	19/20
The pain and redness disappeared	24/25	25/27	19/20
Palpation shows no lumps	25/25	26/27	20/20
Ultrasound examination shows disappearance of abscess echo	15/15	15/16	8/8

### 3.3 Phylogenetic analysis

In clinical testing, the 15 isolates were identified using the Merieux VITEK MS system, with all results reported as *C. kroppenstedtii*. Based on the sequence analysis of *16S rRNA* genes, the 15 isolates are closely related to *C. kroppenstedtii* DSM 44385, with similarity ranging from 99.5 to 99.8% (Supplementary Table 2). The *rpoB* and *fusA* genes of these isolates exhibit homology with *C. kroppenstedtii* DSM 44385, ranging from 94.3 to 96.9% and 95.8 to 99.7%, respectively. Phylogenetic tree analysis of the *rpoB* and *fusA* genes revealed that *C. parakroppenstedtii* accounts for 80% (12/15), *C. pseudokroppenstedtii* accounts for 13.3% (2/15), while *C. kroppenstedtii* comprises only 6.7% (1/15) (Supplementary Figure 1). Previous studies have found that *C. kroppenstedtii* accounts for only minimal proportion of the detected *Corynebacterium* identified in mastitis patients, with *C. parakroppenstedtii* being the most prevalent species, followed by *C. pseudokroppenstedtii*. The combined prevalence of the latter two species is nearly equivalent to the total (Luo et al., 2022; Liu et al., 2024). Our results are consistent with the findings reported in the literature

### 3.4 Drug susceptibility testing

The reason for not grouping the antimicrobial susceptibility testing is that members within the species complex are generally considered to have similar resistance characteristics when there is insufficient understanding of individual members. Therefore, it is recommended to use the same phenotypic testing methods, breakpoints, and interpretative categories (CLSI, 2023). Additionally, it is a small sample size, particularly for *C. pseudokroppenstedtii* and *C. kroppenstedtii*, which does not provide representative drug susceptibility data. Finally, the applicability of the susceptibility testing methods remains to be clarified. Nevertheless, the MIC value for 15 strains against 14 antimicrobial tests are shown in Supplementary Tables 3, 4.

The results of the agar dilution method for testing 14 drugs and the micro broth dilution method for determining daptomycin are shown in Table 3. In the agar dilution method, clindamycin and erythromycin-sensitive strains are derived from the same strain. The rifampicin and ciprofloxacin-resistant strains are the same strain. All other data are completely consistent except for penicillin G and ciprofloxacin, which have partial consistency. While there was a strong concordance between the two methods in terms of phenotypic interpretation results, over 50% of the drug MIC values exhibited significant variability (EA < 85%), indicating that β-NAD may still exert an influence on the test outcomes. For instance, meropenem demonstrated an EA of only 18.75%, despite all results being classified as ‘S’. Two clinical strains displayed ‘S’ to daptomycin in the presence of β-NAD and ‘R’ in its absence, representing an obvious error. In light of the lack of a superior identification method, daptomycin should not be endorsed for antimicrobial susceptibility testing or treatment protocols. Based on our experiences with β-NAD in other assays, its purported “growth promotion” effect on bacteria does not account for daptomycin’s sensitivity profile.

**TABLE 3:** Agar dilution method for testing 14 drugs and micro broth dilution method for determining daptomycins.

		MIC50 (μ g/ml)	MIC90 (μ g/ml)	DSM 44385T (μ g/ml)	Rang (μ g/ml)	EA (%)	Breakpoint (μ g/ml)	Susceptible (%)	Intermediate (%)	Resistant (%)
Cefepime	+	0.06	0.06	0.06	<0.015∼0.06	87.5%	≤1, 2,≥4	100% (15/15)	0	0
	-	0.06	0.06	0.03	<0.015∼0.06			100% (15/15)	0	0
Ceftriaxone	+	0.125	1	0.5	0.015∼1	93.75%	≤1, 2,≥4	100% (15/15)	0	0
	-	0.125	2	0.25	<0.015∼2			100% (15/15)	0	0
Penicillin G	+	0.125	0.5	0.125	<0.015∼1	62.5%	≤0.25, 2,≥4	60%(9/15)	40% (6/15)	0
	-	0.06	0.125	0.03	<0.015∼0.125			100% (15/15)	0	0
Gentamicin	+	<0.015	<0.015	<0.015	0.015	100%	≤0.125, 0.25∼2,≥4	100% (15/15)	0	0
	-	<0.015	<0.015	<0.015	0.015			100% (15/15)	0	0
Meropenem	+	0.015	0.125	0.03	<0.015∼0.25	18.75%	≤0.25, 0.5,≥1	100% (15/15)	0	0
	-	0.06	0.125	0.125	<0.015∼0.25			100% (15/15)	0	0
Erythromycin	+	8	8	0.03	0.06∼8	81.25%	≤0.5, 1,≥2	13.3% (2/15)	0	86.7% (13/15)
	-	8	8	0.125	0.125∼8			13.3% (2/15)	0	86.7% (13/15)
Clindamycin	+	>32	>32	0.06	0.03∼32	100%	≤0.5, 1∼2,≥4	13.3% (2/15)	0	86.7% (13/15)
	-	>32	>32	0.125	0.03∼32			13.3% (2/15)	0	86.7% (13/15)
Doxycycline	+	0.5	1	0.06	<0.03∼1	75%	≤1, 8,≥16	100% (15/15)	0	0
	-	2	2	0.03	<0.03∼2			100% (15/15)	0	0
Linezolid	+	0.25	0.25	0.25	0.125∼0.5	100%	≤2	100% (15/15)	N	0
	-	0.25	0.5	0.25	0.25∼0.5			100% (15/15)	N	0
Ciprofloxacin	+	0.25	2	0.25	0.125∼8	31.25%	≤1, 2,≥4	60% (9/15)	33% (5/15)	7% (1/15)
	-	0.03	2	0.03	0.015∼8			73% (11/15)	20% (3/15)	7% (1/15)
Tetracycline	+	4	4	4	0.03∼4	56.25%	≤1, 8,≥16	100% (15/15)	0	0
	-	2	2	2	0.125∼2			100% (15/15)	0	0
Vancomycin	+	0.125	0.06	0.25	0.125∼0.06	56.25%	≤2	100% (15/15)	N	0
	-	0.25	0.25	0.25	0.25∼0.5			100% (15/15)	N	0
Rifampin	+	0.015	0.015	<0.015	0.015∼32	100%	≤1, 2, ≥4	93%(14/15)	0	7% (1/15)
	-	0.015	0.015	<0.015	0.015∼32			93% (14/15)	0	7% (1/15)
Trimethoprim-Sulfamethoxazole	+	1/19	1/19	1/19	1/19	100%	≤2/38, ≥4/76	100% (15/15)	N	0
	-	1/19	1/19	1/19	1/19			100% (15/15)	N	0
Daptomycin*	+	1	32	16	0.5 ∼>32	N	≤1	80% (12/15)	N	20% (3/15)
	-	1	2	2	0.5 ∼ 4			66.7% (10/15)	N	33.3% (5/15)

“+” indicated 20 mg/mL β-NAD was added; “-” indicates that β-NAD has not been added. Essential agreement (EA): The percentage value where two groups of MIC values do not exceed 1 dilution. The total number of strains in the group without β-NAD was 16 as the denominator, and the number of strains in the group with β-NAD and the group without β-NAD was numerator with MIC value within 1 dilution ratio.

“*”: daptomycin was determined by the microbroth dilution method and calcium ion correction was performed. Other drugs were determined by the AGAR dilution method. N indicates that there is no corresponding setting or no result is provided. The breakpoint was derived from the CLSI micro broth dilution method.

The comparison of the test results between the non-added β-NAD agar dilution method and the micro broth dilution method is shown in Table 4. The SXT micro broth dilution method demonstrated no influence from β-NAD; however, inconsistencies were observed with penicillin G, meropenem, clindamycin, and ciprofloxacin in the micro broth dilution method regardless of the presence of β-NAD. Consequently, it was concluded that this method is not applicable. In contrast, the other nine drugs tested in both the micro broth dilution without the β-NAD group and AGAR dilution without the β-NAD group exhibited complete consistency. Given the inconsistencies associated with adding β-NAD to all drugs except SXT, it is inferred that the EUCAST micro broth dilution procedure is unsuitable for assessing CKC susceptibility tests; furthermore, comparisons regarding differences arising from varying breakpoints are deemed unnecessary.

**TABLE 4:** Comparison between micro broth dilution method and agar dilution method (not added β- NAD).

		Agar dilution method (not added β - NAD)			
**Broth microdilution method**		**Complete consistency**	**Partial consistency**	**Inconsistency**	**Judgment**
Cefepime	+	0	0	100% (16/16)	×
	-	93.75% (15/16)	6.25% (1/16)*	0	√
Ceftriaxone	+	0	0	100% (16/16)	×
	-	100% (16/16)	0	0	√
Penicillin G #	+	0	0	100% (16/16)	×
	-	12.5% (2/16)	81.25% (13/16)	6.25% (1/16)*	×
Gentamicin #	+	81.25% (13/16)*	0	18.75% (3/16)	×
	-	100% (16/16)	0	0	√
Meropenem	+	6.25% (1/16)	18.75% (3/16)	75% (12/16)*	×
	-	12.5% (2/16)	18.75% (3/16)	68.75% (11/16)*	×
Erythromycin	+	81.25% (13/16)	0	18.75% (3/16)*	×
	-	100% (16/16)	0	0	√
Clindamycin #	+	93.75% (15/16)*	0	6.25% (1/16)	×
	-	87.5% (14/16)*	0	12.5% (2/16)	×
doxycycline	+	93.75% (15/16)*	0	6.25% (1/16)	×
	-	100% (16/16)	0	0	√
Linezolid #	+	81.25% (13/16)*	0	18.75% (3/16)	×
	-	100% (16/16)	0	0	√
Ciprofloxacin#	+	25% (4/16)	43.75% (7/16)	31.25% (5/16)*	×
	-	56.25% (9/16)*	31.25% (5/16)	12.5% (2/16)	×
Tetracycline #	+	87.5% (14/16)	0	12.5% (2/16)*	×
	-	100% (16/16)	0	0	√
Vancomycin #	+	68.75% (11/16)	0	31.25% (5/16)*	×
	-	100% (16/16)	0	0	√
Rifampin #	+	6.25% (1/16)	0	93.75% (15/16)*	×
	-	100% (16/16)	0	0	√
Trimethoprim-sulfamethoxazole	+	100% (16/16)	0	0	√
	-	100% (16/16)	0	0	√

“+”indicates the addition of 20 mg/ml β-NAD using the micro broth dilution method;“-” indicates that the micro broth dilution method did not add β-NAD;

“*” indicates the presence of model strains; “#” represents the recommended medication for EUCAST. “inconsistency”, which contains the possibility of “false sensitivity” and “false resistance”, is indicated by “ × ”. “Complete consistency” and “partial consistency” are considered acceptable and are represented by “√”.

### 3.5 Performance of DSM 44385 in micro broth tests

In the AGAR dilution method, when β-NAD was not utilized as a reference, all antibiotics including linezolid, clindamycin, vancomycin, meropenem, cefepime, tetracycline, doxycycline, ceftriaxone, ciprofloxacin, rifampicin and erythromycin in the β-NAD dilution group exhibited false ‘R’ results. Penicillin G also demonstrated a false ‘R’ in both groups; however, gentamicin and SXT showed complete consistency with the AGAR dilution results across both groups. In addition, TW80 has a significant growth-promoting effect, and all test wells of penicillin G grow vigorously, showing resistance (>32 μg/mL).

### 3.6 Drug sensitivity result

Except for daptomycin, the final drug susceptibility test was based on the agar dilution method without adding β-NAD. All clinically isolated strains were sensitive to linezolid, gentamicin, vancomycin, meropenem, cefepime, tetracycline, doxycycline, penicillin G, ceftriaxone, and SMZ-TMP. The resistance rate to rifampicin and ciprofloxacin was 7% (1/15), and the resistance rate to erythromycin and clindamycin was 86.7% (13/15). DSM 44385 is sensitive to all drugs. Daptomycin is not reported.

## 4 Discussion

*C. kroppenstedtii* was first isolated and described in sputum in 1998, and confirmed that it was closely associated with mastitis in non-lactating women. However, recent research has confirmed the existence of the CKC and has challenged the correlation between *C. kroppenstedtii* and mastitis (Huang et al., 2024; Liu et al., 2024). In earlier case reports, the detection of CKC in the mammary glands of white women was defined as uncommon cases (Le Flèche-Matéos et al., 2012). There is no accurate statistical data on the detection rate of CKC in mastitis in the Chinese Mainland. Still, many CKC cases are reported in the literature in a single area (Zhang et al., 2023). Combined with the huge population base, CKC is a common bacterium in non-lactating mastitis in Chinese Mainland. CKC has garnered significant attention due to its prevalent association with non-lactational mastitis. Our mammary pus samples were collected in the operating room, revealing colony counts of CKC ranging from a dozen to a hundred. Some patients exhibited multiple detections or bilateral involvement simultaneously, with all cases demonstrating simple bacterial growth without any concomitant polymicrobial infections. This supports the notion of CKC as a potential pathogen. The standardization of quantitative inoculation of abscess samples remains challenging and complex in practice. CKC cultured from deep sterile tissues, regardless of the quantity, still possesses a high potential for pathogenicity (Paviour et al., 2002).

The pathological changes of mastitis have very similar morphology. In many cases, cystic neutrophilic granulomatous mastitis (CNGM) has obvious pathological and clinical overlap with other granulomatous mastitis (GM). In addition, there is terminology confusion between GM, GLM, idiopathic granulomatous mastitis (IGM) and CNGM in different reports (Wu and Turashvili, 2020), which makes it difficult for pathologic classification and extract effective information from the literature. CNGM was once considered to be the most closely related pathological type of *Corynebacterium* infection (Yuan et al., 2022), but this view was not supported by some studies (D’Alfonso et al., 2015). Some researchers suggested that the pattern may represent a continuum of IGM (Aljawder et al., 2023). For these reasons, our case was not classified by CNGM, and the concomitant diagnosis also demonstrated the overlap of pathological changes in mastitis. We believe that in the dynamic evolution of inflammation, it is not so important to find a characteristic association between pathological classification and CKC infection, the focus is on whether it is infectious or not. When positive etiological evidence is obtained, targeted treatment can lead to good treatment expectations for simple infectious mastitis and ease the complexity of disease development.

The clinical data of patients in the CKC culture-positive group (group A) and culture-negative group (group B) were compared, and it was found that the CPR, WBC and NEUT in group A were significantly higher than those in group B (*P* < 0.05), suggesting that in the same case of severe abscess, CKC infection can cause more obvious inflammatory response, which also indicates that CKC does play an inflammatory pathological role in mastitis. The level of prolactin in group A and group B was significantly different (*P* < 0.001), which further confirmed the close association between increased prolactin and CKC infection. Our grouping method found A significant difference in the age of first delivery between group A and group B (*P* < 0.001), suggesting that elderly primipara combined with high levels of prolactin are high-risk groups for CKC infection.

Prolactin seems to play a nodal role in CKC infection. Causes such as pituitary tumor, drug-induced hyperprolactin, sex hormone disorder, and thyroid hormone abnormality eventually act on mammary ducts in the form of elevated prolactin, causing inflammation and increasing the risk of CKC infection (Zeng et al., 2023). Typical cases are as follows: In a patient with craniopharyngioma, GM, and elevated prolactin, the inflammatory breast lesions completely subsided after craniopharyngioma resection and prolactin levels returned to normal, indicating that hyperprolactinemia caused by intracranial lesions may be the direct cause of GM (Nikolaev et al., 2016). Moreover, psychotropic medications that may lead to hyperprolactinemia are significant risk factors for CKC (Wong et al., 2017).

The information about the correlation between tobacco and mastitis is quite confusing. A study showed that NPM has been identified as a smoker’s disease (Risager and Bentzon, 2010). In contrast, some studies indicate that non-smokers are more prone to recurrent and long-term infections (Co et al., 2018). Furthermore, additional research suggests that IGM and GM are not associated with smoking (Al-Khaffaf et al., 2008; Zeng et al., 2023). This inconsistency underscores the complexity of understanding the relationship between tobacco use and mastitis. There were no smokers in our case, and the high prevalence of smoking among men in China leads to high exposure of women to second-hand smoke, the effect of which on mastitis is difficult to control. Tobacco surveys should include, at a minimum, daily tobacco consumption and the number of years of smoking history. We speculate that the smoking habit may imply other bad lifestyle habits and/or more negative emotions, which may lead to decreased immunity and an increased chance of endocrine disruption, thereby increasing the incidence of mastitis. It is suggested that in addition to considering the direct harm of tobacco, information on indirect potential health damage should also be considered in subsequent studies.

Susceptibility test is an important basis to guide the use of antibiotics, but EUCAST and CLSI lack specific Settings for CKC susceptibility test, so we doubt the results of CKC susceptibility test reported in the literature. In the preliminary experiments of this study, β-NAD and TW80 were found to have an important effect on the drug susceptibility test of CKC, especially for the micro broth dilution method. According to EUCAST and CLSI documents, different addition criteria will give opposite conclusions (R and S) for susceptibility tests of certain strains or certain classes of drugs. AGAR dilution is generally considered to be the gold standard of drug susceptibility testing. We tested 14 antibiotics with AGAR dilution, and although the final interpretation of 13 antibiotics was “Complete consistency”, the EA value reflected the potential effect of β-NAD on more than half of the drugs (EA < 85%). The presence or absence of β-NAD had a decisive influence on the results of the micro broth dilution test, and while the growth-promoting effect of β-NAD on CKC explained most of the resistance, two clinical strains of daptomycin showed the opposite, although the test was repeated.

TW80 has a very obvious growth-promoting effect on CKC in a liquid environment. Penicillin G was randomly selected for testing in this study to verify the effect of TW80 on drug susceptibility tests, and all results showed “drug resistance”. However, in the recently reported drug susceptibility test of CKC in large samples, the resistance rate of penicillin after 0.5% TW80 was added by micro broth dilution method was only 7.8% (Zhang et al., 2023), which was significantly different from our drug-resistance testing results. The reported results were more similar to our micro broth dilution method without adding β-NAD, both the “intermediate” values were 86.6%. The powerful growth-promoting effect of TW80 has been confirmed (Hieken et al., 2016; Tauch et al., 2016) which is more consistent with the performance of penicillin total resistance after adding 0.5% TW80 in our study. In the drug susceptibility test of the commercial micro broth dilution method for DSM 44385, ceftriaxone is “I” and cefepime is “R” (Luo et al., 2022), the corresponding test results of our micro broth dilution method are “S” and “I” respectively, and the test results of AGAR dilution method are “S”. Since the detailed ingredient list of the commercial reagent is not obtained, the reasons for the differences were not analyzed.

In the absence of a uniform standard, we did not conduct additional evaluation work, such as comparison with other reported drug susceptibility results, the effect of β-NAD and TW80 on E-test, the effect of TW80 on more drugs in micro broth dilution, and resistance gene testing. Furthermore, following multiple passages, CKC demonstrates rapid and robust growth on standard blood agar plates. In this study, CKC was subcultured for more than three passages in the laboratory, achieving satisfactory growth within 24 h on conventional BAP and yielding consistent results within 36 h during drug susceptibility testing. However, for strains of CKC that have undergone fewer than three passages or primary isolation, the appropriateness of the time settings in drug susceptibility tests remains to be validated. While AGAR dilution test outcomes can be considered reliable indicators of drug sensitivity, the intricate procedure is not conducive to routine clinical application. The micro broth dilution method stands as the most prevalent approach for drug susceptibility testing in clinical settings; thus, enhancements to standardized testing protocols are warranted.

Our inclusion criteria excluded cases of tuberculosis and staphylococcal mastitis, which may result in sample distribution bias and substantial discrepancies compared to other research data (Costa Morais Oliveira et al., 2021; Stevenson et al., 2022). In this study, the detection rate of CKC culture in non-lactation mastitis was as high as 47.3% (26/55), which was related to the following factors. These cases were all patients who underwent breast segmentation resection and surgical discharge of pus and had severe inflammatory lesions, and CKC had sufficient growth time to increase the probability of detection. The special training in CKC mastitis gives laboratory technicians sufficient knowledge of it. In the culture procedure, a BAP plate coated with 1% TW80 was added to the breast pus samples, which significantly improved the detection rate of CKC and shortened the culture time. With mass spectrometer identification ability, can quickly and accurately distinguish from other Corynebacterium. All patients were Han, and no other races were included, so racial differences could not be ruled out. In addition, missing culture records in 22 cases resulted in statistical bias.

In this study, all patients received intravenous infusions of cefuroxime sodium and/or levofloxacin before surgery to mitigate the risk of surgical wound infections. Additionally, bromocriptine was administered to patients exhibiting elevated serum prolactin levels or residual milk in the surgical area. Routine drug susceptibility testing was not performed for cases where CKC was identified. Beyond the limitations associated with the drug susceptibility testing procedure, the pathogenic mechanisms underlying CKC remain unclear, and there is no clinical consensus regarding its targeted treatment approach. Based on individual patient conditions, several of these herbal remedies were selected for combination therapy through decoction and/or external application. TCM posits that these substances are advantageous for wound healing and the reduction of inflammation, with several having been substantiated through research (Xue et al., 2020). In a telephone follow-up conducted 12 months post-discharge, 6% (4/72) of patients did not exhibit satisfactory recovery, leaving long-term efficacy uncertain. This study found that CKC demonstrated notable sensitivity to most antibiotics; however, the unique structure of the mammary gland rendered lipophilic antibiotics more selective and was deemed responsible for the suboptimal effectiveness of β-lactam and fluoroquinolone agents (Dobinson Hazel et al., 2015). Treatment regimens incorporating rifampicin have been shown to significantly reduce recurrence rates among CKC infected patients; nevertheless, further evidence and prospective studies are warranted (Li et al., 2023). The elevated resistance rates observed with erythromycin and clindamycin alongside the emergence of rifampicin-resistant strains underscore the critical need for drug sensitivity testing. Rifampicin should be utilized judiciously, particularly in light of the current global resurgence in tuberculosis infections.

In clinical practice, the three species of bacteria are sensitive to most of antibiotics, with no significant differences observed in result. The precise identification of CKC strains is primarily significant for epidemiological purposes. Due to the minimal genetic differences between CKC species and the lack of established genotype consistency, accurate strain identification proves to be challenging. Whole-genome sequencing can assist in distinguishing species within CKC (Huang et al., 2024). However, it is evident that such technique can only be implemented in advanced laboratories. Recently, a novel mass spectrometry peaks (MSP) for *C. parakroppenstedtii* using Bruker MALDI-TOF MS provides a feasible approach for more routine clinical laboratories to achieve accurate identifications (Xiao and Zhao, 2024).

This study engages in a comprehensive discussion regarding the methodology for assessing drug susceptibility of CKC, and proposes specific modifications to the clinical drug susceptibility testing procedures for this organism. Currently, all available literature consists of retrospective studies characterized by incomplete sample data and biased sample distributions, which result in varying degrees of limitations in their conclusions. Nevertheless, from the standpoint of clinical microbial etiology, it can be posited that the first two postulates of Koch’s Postulates have been substantiated; however, further closed-loop validation through relevant animal models is required for the third and fourth postulates, alongside an expectation for more robust prospective clinical studies.

## Data Availability

The original contributions presented in the study are publicly available. This data can be found here: GenBank, accession numbers PQ538763 - PQ538777 and PQ660463 - PQ660492.

## References

[B1] Aljawder A. A. Li J. J. Ng J. K. Chan R. C. Lui P. C. Poon I. K. (2023). Idiopathic granulomatous mastitis and cystic neutrophilic granulomatous mastitis: Two sides of the same coin or distinct entities? *Pathology* 55 335–341. 10.1016/j.pathol.2022.09.005 36503636

[B2] Al-Khaffaf B. Knox F. Bundred N. J. (2008). Idiopathic granulomatous mastitis: A 25-year experience. *J. Am. Coll. Surg.* 206 269–273. 10.1016/j.jamcollsurg.2007.07.041 18222379

[B3] Busse H. J. Kleinhagauer T. Glaeser S. P. Spergser J. Kämpfer P. Rückert C. (2019). Classification of three corynebacterial strains isolated from the Northern Bald Ibis (Geronticus eremita): Proposal of Corynebacterium choanae sp. nov., Corynebacterium pseudopelargi sp. nov., and Corynebacterium gerontici sp. nov. *Int. J. Syst. Evol. Microbiol.* 69 2928–2935. 10.1099/ijsem.0.003580 31310200

[B4] CLSI (2023). *Performance Standards for Antimicrobial Susceptibility Testing. CLSI supplement M100*, 33rd Edn. Wayne, PA: Clinical and Laboratory Standards Institute.

[B5] Co M. Cheng V. C. Wei J. Wong S. C. Chan S. M. Shek T. (2018). Idiopathic granulomatous mastitis: A 10-year study from a multicentre clinical database. *Pathology* 50 742–747. 10.1016/j.pathol.2018.08.010 30389215

[B6] Costa Morais Oliveira V. Cubas-Vega N. López Del-Tejo P. Baía-da-Silva D. C. Araújo Tavares M. Picinin Safe I. (2021). Non-lactational infectious Mastitis in the Americas: A systematic review. *Front. Med.* 8:672513. 10.3389/fmed.2021.672513 34422853 PMC8378399

[B7] D’Alfonso T. M. Moo T. A. Arleo E. K. Cheng E. Antonio L. B. Hoda S. A. (2015). Cystic neutrophilic granulomatous mastitis: Further characterization of a distinctive histopathologic entity not always demonstrably attributable to corynebacterium infection. *Am. J. Surg. Pathol.* 39 1440–1447. 10.1097/PAS.0000000000000479 26200100

[B8] Dobinson Hazel C. Anderson Trevor P. Chambers Stephen T. Doogue Matthew P. Seaward L. Werno Anja M. (2015). Antimicrobial treatment options for granulomatous mastitis caused by corynebacterium species. *J. Clin. Microbiol.* 53 2895–2899. 10.1128/JCM.00760-15 26135858 PMC4540898

[B9] Hieken T. J. Chen J. Hoskin T. L. Walther-Antonio M. Johnson S. Ramaker S. (2016). The microbiome of aseptically collected human breast tissue in benign and malignant disease. *Sci. Rep.* 6:30751. 10.1038/srep30751 27485780 PMC4971513

[B10] Huang Y. Song M. H. Li S. G. Yu Shen H. Qu P. H. Zhang D. F. (2024). Preliminary comparative genomics analysis among Corynebacterium kroppenstedtii complex necessitates a reassessment of precise species associated with mastitis. *J. Appl. Microbiol.* 135:lxad314. 10.1093/jambio/lxad314 38130215

[B11] Khamis A. Raoult D. La Scola B. (2004). rpoB gene sequencing for identification of Corynebacterium species. *J. Clin. Microbiol.* 42 3925–3931. 10.1128/JCM.42.9.3925-3931.2004 15364970 PMC516356

[B12] Le Flèche-Matéos A. Berthet N. Lomprez F. Arnoux Y. Le Guern A. S. Leclercq I. (2012). Recurrent breast abscesses due to Corynebacterium kroppenstedtii, a human pathogen uncommon in caucasian women. *Case Rep. Infect. Dis.* 2012:120968. 10.1155/2012/120968 23008788 PMC3449112

[B13] Li S. Huang Q. Song P. Han X. Liu Z. Zhou L. (2023). Clinical characteristics and therapeutic strategy of granulomatous mastitis accompanied by Corynebacterium kroppenstedtii: A retrospective cohort study. *BMC Womens Health* 23:388. 10.1186/s12905-023-02509-7 37491234 PMC10369769

[B14] Liu R. Luo Z. Dai C. Wei Y. Yan S. Kuang X. (2024). Corynebacterium parakroppenstedtii secretes a novel glycolipid to promote the development of granulomatous lobular mastitis. *Signal Transduct. Targeted Ther.* 9:292. 10.1038/s41392-024-01984-0 39428541 PMC11491465

[B15] Luo Q. Chen Q. Feng J. Zhang T. Luo L. Chen C. (2022). Classification of 27 Corynebacterium kroppenstedtii-like isolates associated with mastitis in China and descriptions of C. parakroppenstedtii sp. nov. and C. pseudokroppenstedtii sp. nov. *Microbiol. Spectr.* 10:e0137221. 10.1128/spectrum.01372-21 35289670 PMC9045094

[B16] Nikolaev A. Blake C. N. Carlson D. L. (2016). Association between hyperprolactinemia and granulomatous mastitis. *Breast J.* 22 224–231. 10.1111/tbj.12552 26705962

[B17] Paviour S. Musaad S. Roberts S. Taylor G. Taylor S. Shore K. (2002). Corynebacterium species isolated from patients with mastitis. *Clin. Infect. Dis.* 35 1434–1440. 10.1086/344463 12439810

[B18] Que Y. Shi J. Zhang Z. Sun L. Li H. Qin X. (2024). Ion cocktail therapy for myocardial infarction by synergistic regulation of both structural and electrical remodeling. *Exploration* (Beijing, China). 4:20230067. 10.1002/EXP.20230067 PMC11189571 38939858

[B19] Risager R. Bentzon N. (2010). [Smoking and increased risk of mastitis]. *Ugeskrift Laeger.* 172 2218–2221. 20727287

[B20] Stevenson D. R. Das S. Lambourne J. Ledwidge S. F. Johnson L. Rosmarin C. (2022). Corynebacterium kroppenstedtii breast abscesses in context, a retrospective cohort study. *J. Med. Microbiol.* 71. 10.1099/jmm.0.001616 36748506

[B21] Tauch A. Fernández-Natal I. Soriano F. (2016). A microbiological and clinical review on Corynebacterium kroppenstedtii. *Int. J. Infect. Dis.* 48 33–39. 10.1016/j.ijid.2016.04.023 27155209

[B22] Weisburg W. G. Barns S. M. Pelletier D. A. Lane D. J. (1991). 16S ribosomal DNA amplification for phylogenetic study. *J. Bacteriol.* 173 697–703. 10.1128/jb.173.2.697-703.1991 1987160 PMC207061

[B23] Wong S. C. Poon R. W. Chen J. H. Tse H. Lo J. Y. Ng T. K. (2017). Corynebacterium kroppenstedtii is an emerging cause of mastitis especially in patients with psychiatric illness on antipsychotic medication. *Open Forum Infect. Dis.* 4:ofx096. 10.1093/ofid/ofx096 28852671 PMC5570011

[B24] Wu J. M. Turashvili G. (2020). Cystic neutrophilic granulomatous mastitis: An update. *J. Clin. Pathol.* 73 445–453. 10.1136/jclinpath-2019-206180 32094275

[B25] Xiao N. Zhao X. Y. (2024). Clinical identification of two novel C. kroppenstedtii-like species isolated as pathogens of granulomatous lobular mastitis. *Pathogens (Basel, Switzerland)* 13:880. 10.3390/pathogens13100880 39452751 PMC11514597

[B26] Xue J. X. Ye B. Liu S. Cao S. H. Bian W. H. Yao C. (2020). Treatment efficacy of Chuang Ling Ye, a traditional Chinese herbal medicine compound, on idiopathic granulomatous mastitis: A randomized controlled trial. *Evid. Based Complement. Altern. Med.* 2020:6964801. 10.1155/2020/6964801 32714413 PMC7341429

[B27] Yuan Q. Q. Xiao S. Y. Farouk O. Du Y. T. Sheybani F. Tan Q. T. (2022). Management of granulomatous lobular mastitis: An international multidisciplinary consensus (2021 edition). *Milit. Med. Res.* 9:20. 10.1186/s40779-022-00380-5 PMC9040252 35473758

[B28] Zeng Y. Zhang D. Fu N. Zhao W. Huang Q. Cui J. (2023). Risk factors for granulomatous mastitis and establishment and validation of a clinical prediction model (Nomogram). *Risk Manag. Healthc. Policy* 16 2209–2222. 10.2147/RMHP.S431228 37881167 PMC10596285

[B29] Zhang Q. Wu S. Song P. Liu Y. Ding L. Shi Q. (2023). Antibiotic resistance and resistance mechanism of Corynebacterium kroppenstedtii isolated from patients with mastadenitis. *Eur. J. Clin. Microbiol. Infect. Dis.* 42 525–528. 10.1007/s10096-023-04558-0 36847927

