# Supplementary material for: Clinical review and drug sensitivity test of Corynebacterium kroppenstedtii complex isolates in non-lactating patients with severe mastitis
Source: Front Microbiol. 2024 Dec 23;15:1501204. doi: 10.3389/fmicb.2024.1501204 (PMC11701807; doi:10.3389/fmicb.2024.1501204)
Supplement: Supplementary file 1 [file Data_Sheet_1.docx]

Supplementary Material

**Supplementary Table 1**: Primers used for amplification of the *16S rRNA, rpoB,* and *fusA* gene.

| Primer | Primer sequence (5′-3′) |
| --- | --- |
| *16S rRNA*-27F | AGAGTTTGATCMTGGCTCAG |
| *16S rRNA* -1492R | GGTTACCTTGTTACGACTT |
| *rpoB*-C2700F | CGWATGAACATYGGBCAGGT |
| *rpoB*-C3130R | TCCATYTCRCCRAARCGCTG |
| *fusA*-401F | GCTTCGTSAACAAGATGGAC |
| *fusA*-1516R | RGTCTGCTTCTTGTGGGTG |

| **Supplementary Table 2**: Identification results of CKC isolates | | | |  |  |
| --- | --- | --- | --- | --- | --- |
| Strain | *16S rRNA* gene | *rpoB* gene | *fusA* gene | *16S rRNA* gene GenBank accession |  |
|  | %Identity | %Identity | %Identity |  |  |
| 22007800 | 99.6 | 94.7 | 97.7 | PQ538763 |  |
| 22006630 | 99.6 | 95.6 | 97.5 | PQ538764 |  |
| 22007791 | 99.6 | 96.9 | 97.2 | PQ538765 |  |
| 21009998 | 99.6 | 95.4 | 97.6 | PQ538766 |  |
| 22007452 | 99.6 | 95.4 | 97.5 | PQ538767 |  |
| 22008060 | 99.6 | 95.4 | 97.4 | PQ538768 |  |
| 22006520 | 99.6 | 95.1 | 97.5 | PQ538769 |  |
| 22007792 | 99.6 | 95.4 | 97.5 | PQ538770 |  |
| 22001146 | 99.6 | 95.4 | 97.6 | PQ538771 |  |
| 21010366 | 99.6 | 95.1 | 97.6 | PQ538772 |  |
| 22010037 | 99.7 | 95.4 | 97.3 | PQ538773 |  |
| 22008376 | 99.6 | 95.4 | 97.7 | PQ538774 |  |
| 22008056 | 99.5 | 95.1 | 97.6 | PQ538775 |  |
| 23000938 | 99.8 | 95.4 | 97.4 | PQ538776 |  |
| 22010946 | 99.6 | 94.3 | 95.8 | PQ538777 |  |

| MIC (μg/mL) | 0.015 | 0.03 | 0.06 | 0.125 | 0.25 | 0.5 | 1 | 2 | 4 | 8 | 32 | 1/19 |
| --- | --- | --- | --- | --- | --- | --- | --- | --- | --- | --- | --- | --- |
| Linezamine |  |  |  |  | 9+1^a^ | 3+1^a^+1^b^ |  |  |  |  |  |  |
| Clindamycin |  |  |  | 3 |  |  |  |  |  |  | 9+2^a^+1^b^ |  |
| Gentamycin | 12+2^a^+1^b^ |  |  |  |  |  |  |  |  |  |  |  |
| Vancomycin |  |  |  |  | 11+2^a^+1^b^ | 1 |  |  |  |  |  |  |
| Meropenem | 1 |  | 7 | 2+2^a^+1^b^ | 2 |  |  |  |  |  |  |  |
| Rifampicin | 11+2^a^+1^b^ |  |  |  |  |  |  |  |  | 1 |  |  |
| Erythromycin |  |  |  | 2 |  |  |  |  | 1^a^ | 10+1^a^+1^b^ |  |  |
| Cefepime | 3 | 4 | 4+2^a^+1^b^ |  |  | 1 |  |  |  |  |  |  |
| Tetracycline |  |  | 1^a^+1^b^ | 3 |  | 1 | 1 | 7+1^a^ |  |  |  |  |
| Doxycycline |  | 4+1^a^+1^b^ |  |  |  |  |  | 8+1^a^ |  |  |  |  |
| SXT |  |  |  |  |  |  |  |  |  |  |  | 12+2^a^+1^b^ |
| Ciprofloxacin | 3 | 5 |  |  |  | 1 | 2^a^ | 2+1^a^ |  | 1 |  |  |
| Penicillin G | 1 | 3 | 5 | 3+2^a^+1^b^ |  |  |  |  |  |  |  |  |
| Ceftriaxone | 1 |  | 4 | 2+1^a^ | 3+1^b^ | 1^a^ | 1 | 1 |  |  |  |  |

**Supplementary Table 3**: Distribution of the number of strains with different MIC values from the sensitivity tests of isolates against 14 antibiotics (Agar, without β-NAD).

"a" represents *C. pseudokroppenstedtii*; "b" represents *C. kroppenstedtii*; The unmarked one represents *C. parakroppenstedtii.*

| MIC (μg/mL) | 0.015 | 0.03 | 0.06 | 0.125 | 0.25 | 0.5 | 1 | 2 | 4 | 8 | 32 | 1/19 |
| --- | --- | --- | --- | --- | --- | --- | --- | --- | --- | --- | --- | --- |
| Linezamine |  |  | 3 | 8+2^a^+1^b^ | 1 |  |  |  |  |  |  |  |
| Clindamycin | 1 | 1 |  |  | 1^a^+1^b^ |  |  |  |  |  | 10+1^a^ |  |
| Gentamycin | 6 | 3+2^a^+1^b^ | 3 |  |  |  |  |  |  |  |  |  |
| Vancomycin | 2 |  |  |  |  | 10+1^a^ | 1^a^+1^b^ |  |  |  |  |  |
| Meropenem |  |  |  |  | 2 | 2+1^a^ | 4+1^a^+1^b^ | 1 | 3 |  |  |  |
| Rifampicin | 11+2^a^+1^b^ |  |  |  |  |  |  |  |  | 1 |  |  |
| Erythromycin |  |  | 2 |  |  |  |  |  |  | 10+2^a^+1^b^ |  |  |
| Cefepime |  | 1 | 5 | 4 | 2+2^a^+1^b^ |  |  |  |  |  |  |  |
| Tetracycline |  |  |  | 1 | 2+1^a^+1^b^ | 1 |  | 1 | 6+1^a^ | 1 |  |  |
| Doxycycline |  |  | 1+1^a^ | 2+1^b^ |  |  | 3 | 6+1^a^ |  |  |  |  |
| SXT |  |  |  |  |  |  |  |  |  |  |  | 12+2^a^+1^b^ |
| Ciprofloxacin |  |  |  | 8 |  |  |  | 2 | 2+1^a^+1^b^ | 1^a^ |  |  |
| Penicillin G |  |  |  | 1 | 3 | 5+1^a^ | 2+1^a^+1^b^ | 1 |  |  |  |  |
| Ceftriaxone |  | 1 |  | 7 | 1 | 1+1^b^ | 2^a^ | 2 |  |  |  |  |

**Supplementary Table 4**: Distribution of the number of strains with different MIC values from the sensitivity tests of isolates against 14 antibiotics (Broth, without β-NAD).

"a" represents *C. pseudokroppenstedtii*; "b" represents *C. kroppenstedtii*; The unmarked one represents *C. parakroppenstedtii*

*.*


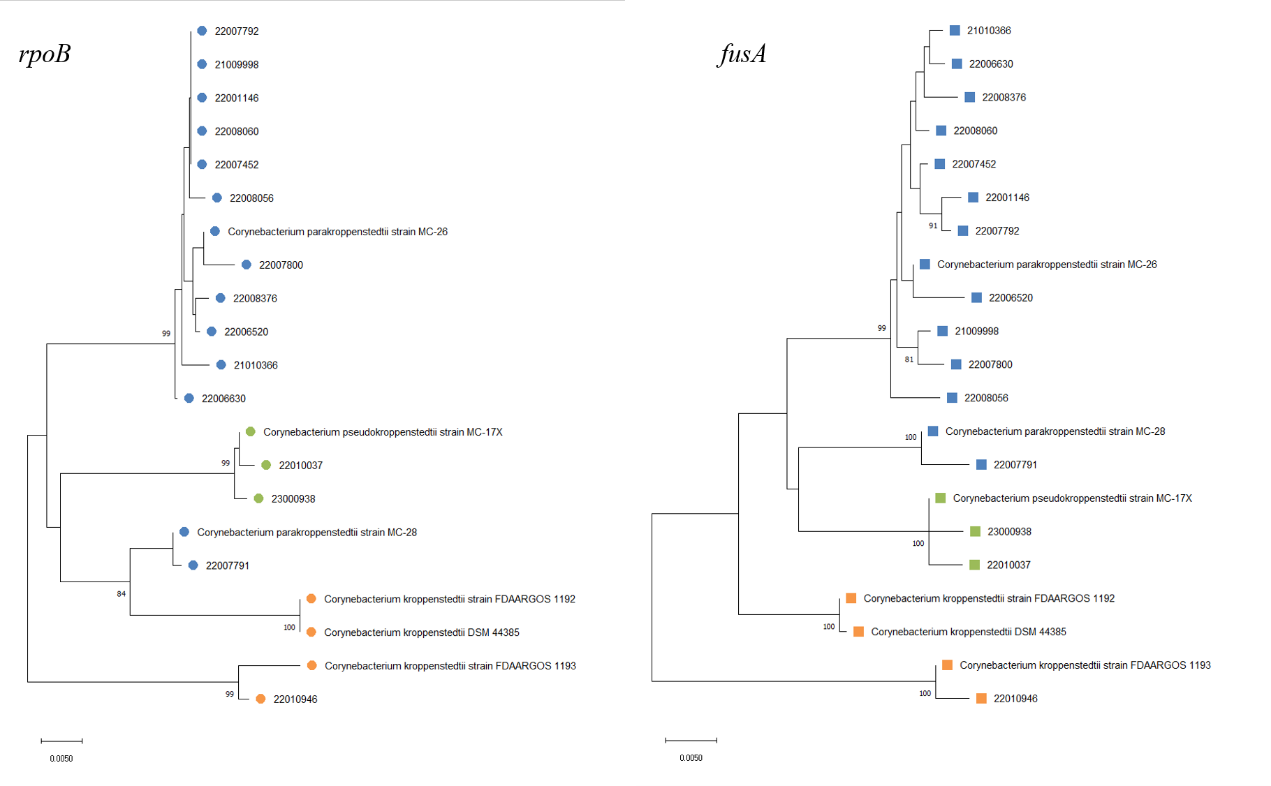


**Supplementary Figure 1.** The neighbor-joining tree constructed based on *rpoB* and *fusA* gene sequences shows the evolutionary relationships of 15 CKC strains with the closely related species. The figure displays bootstrap values calculated from 1,000 repetitions.
